# Ten-Year Follow-Up of Patients Treated with Fecal Microbiota Transplantation for Recurrent *Clostridioides difficile* Infection from a Randomized Controlled Trial and Review of the Literature

**DOI:** 10.3390/microorganisms9030548

**Published:** 2021-03-06

**Authors:** R. E. Ooijevaar, E. van Nood, A. Goorhuis, E. M. Terveer, J. van Prehn, H. W. Verspaget, Y. H. van Beurden, M. G. W. Dijkgraaf, J. J. Keller

**Affiliations:** 1 Department of Gastroenterology and Hepatology, Amsterdam University Medical Centers, VU University Medical Center, De Boelelaan 1117, 1081 HV Amsterdam, The Netherlands; y.vanbeurden@amsterdamumc.nl; 2 Department of Medical Microbiology and Infection Control, Amsterdam University Medical Centers, VU University Medical Center, De Boelelaan 1117, 1081 HV Amsterdam, The Netherlands; 3 Department of Medical Microbiology and Infectious Diseases, Erasmus Medical Center, Doctor Molewaterplein 40, 3015 GD Rotterdam, The Netherlands; e.vannood@erasmus.mc.nl; 4 Department of Infectious Diseases, Amsterdam University Medical Centers, Academic Medical Center, Meibergdreef 9, 1105 AZ Amsterdam, The Netherlands; a.goorhuis@amsterdamumc.nl; 5 Department of Medical Microbiology, Leiden University Medical Center, Albinusdreef 3, 2333 AZ Leiden, The Netherlands; e.m.terveer@lumc.nl (E.M.T.); j.vanprehn@lumc.nl (J.v.P.); 6 Biobank LUMC, Leiden University Medical Center, Albinusdreef 3, 2333 AZ Leiden, The Netherlands; h.w.verspaget@lumc.nl; 7 Department of Epidemiology and Data Science, Amsterdam University Medical Centers, University of Amsterdam, Amsterdam Public Health, Meibergdreef 9, 1105 AZ Amsterdam, The Netherlands; m.g.dijkgraaf@amsterdamumc.nl; 8 Department of Gastroenterology and Hepatology, Leiden University Medical Center, Albinusdreef 3, 2333 AZ Leiden, The Netherlands; 9 Department of Gastroenterology and Hepatology, Haaglanden Medical Center, Postbus 432, 2501 CK The Hague, The Netherlands

**Keywords:** *Clostridioides difficile* infection, fecal microbiota transplantation, FMT, long-term, follow-up, adverse events

## Abstract

Fecal microbiota transplantation (FMT) has become a well-established treatment for recurrent *Clostridioides difficile* infection (rCDI). While short-term outcomes and adverse events relating to FMT have been well documented, there still is a paucity of data with regard to long-term safety. In this report, we describe the long-term follow-up of the prospective cohort of the first randomized controlled trial of FMT for rCDI, and review the existing literature. A total of 34 patients were treated with FMT for rCDI. Seven patients were still alive after a follow-up of more than 10 years and three patients were lost to follow-up. None of the 34 patients had experienced a new-onset autoimmune, gastrointestinal, or malignant disorder during follow-up. We did not find any deterioration or amelioration of pre-existing medical conditions. Furthermore, no deaths directly attributable to FMT could be identified. These findings are in accordance with the data in available literature. In conclusion, no long-term adverse events or complications directly attributable to FMT were found in our prospective cohort. Review of the available literature does not point to long-term risks associated with FMT in this elderly population, provided that carefully screened fecal suspensions are being used. No firm conclusion on the long-term safety of FMT in younger patients could be drawn.

## 1. Introduction

A disturbance of the gut microbiota, often caused by broad-spectrum antibiotics, can result in the germination of *Clostridioides difficile* spores, toxin production, and subsequent *C. difficile* infection (CDI). Recurrence rates of CDI after antibiotics directed towards *C. difficile* remain high (~25%), and increase with each subsequent episode [1]. For patients with recurrent (r)CDI, fecal microbiota transplantation (FMT) is an effective and established treatment [2,3,4], restoring the perturbed and depleted gut microbiota of patients by infusing a balanced and healthy donor (fecal) microbiota preparation [5,6,7,8].

With the implementation of FMT as a treatment for rCDI and its evaluation as a treatment modality in clinical trials for other conditions, the short-term outcome and safety of FMT has been well documented [9]. The most commonly reported immediate adverse reactions following FMT are mild and transient, consisting of abdominal pain, bloating, diarrhea, constipation, and nausea and occur in roughly 29% of patients [10,11]. In addition, procedure (endoscopy)-related serious adverse events may occur rarely, such as perforation, gastrointestinal bleeding, and aspiration. Importantly, only very few serious infectious complications have been described. However, the report of multi-drug resistant Gram-negative sepsis after FMT in neutropenic patients underlines the need for rigorous screening of donors and their respective feces to assure the safety for FMT recipients [12,13]. Whereas short-term safety appears established, there is a paucity of data pertaining to long-term adverse reactions that are possibly attributable to FMT. Concerns have been raised about potential unknown long-term effects of FMT, such as systemic autoimmune, gastrointestinal inflammatory disorders or neoplasia, even though donors are carefully selected as not having any condition associated with a perturbed microbiota [12,14]. In this report, we describe the 10-year follow-up of rCDI patients who were previously treated by FMT in a randomized clinical trial [7]. In addition, a literature search was performed to summarize the available data on long-term follow-up after FMT for the treatment of rCDI.

## 2. Patients and Methods

### 2.1. Patients

In a randomized controlled trial (FECAL trial, Netherlands Trial Register number NTR1177), conducted between 2008 and 2010, 43 patients with rCDI were randomized to receive: (A) FMT preceded by 4 days of vancomycin 500 mg q.i.d. and a bowel lavage (intervention group); (B) 14 days of vancomycin 500 mg q.i.d. (control group); or (C) 14 days of vancomycin 500 mg q.i.d. preceded by a bowel lavage (control group). FMT was performed by infusing a donor fecal suspension through a duodenal tube [7]. Patients with compromised immunity because of recent chemotherapy, those suffering from HIV with a CD4 count below 240, those with a prolonged use of prednisolone (>60 mg per day), those with a need for ICU admission, or those who were pregnant were excluded from the trial.

**Figure 1:**
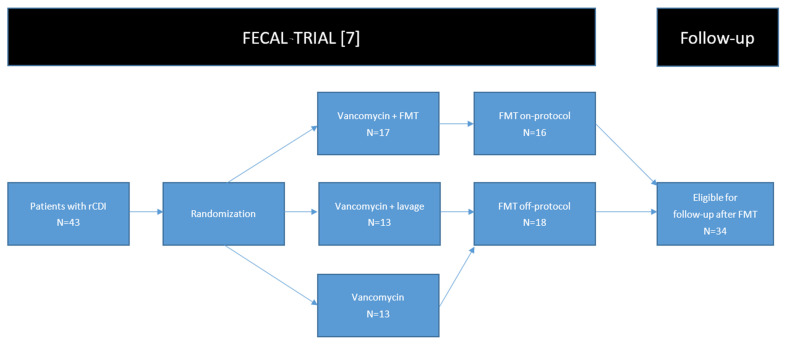
A flowchart of inclusion of patients. One patient randomized to receive FMT dropped out of the trial before receiving FMT, and was therefore not eligible for follow-up. Abbreviations: FMT: fecal microbiota transplantation, rCDI: recurrent *Clostridioides difficile* infection.

After an interim analysis, the trial was stopped prematurely due to an overwhelming difference in efficacy favoring FMT. Seventeen patients were randomized to receive FMT, of whom 16 were treated. In total, 26 patients were assigned to antibiotic treatment (control groups), of whom 19 suffered from a new episode of rCDI after treatment. Of those 19 patients with rCDI after antibiotic treatment, 18 patients received FMT off-protocol (Figure 1). The protocol was approved by the Medical Ethics Committee, and all patients had given written informed consent for inclusion in the trial, and a separate approval for long-term follow-up for more than 10 years.

### 2.2. Collection of Follow-Up Data

Of the included patients who had undergone FMT as a study treatment and those randomized to antibiotic treatment who received FMT off-protocol after antibiotic failure, long-term follow-up data about the recurrence of CDI, antibiotic use, general health, hospitalization, and mortality were collected via a structured telephone interview combined with a review of hospital records, if available. A recurrence was defined as a new microbiologically proven episode of CDI. Participants were contacted at two different timepoints—approximately two and ten years after FMT. Primary healthcare physicians were contacted if a patient was untraceable or unable to provide the information. Cause of death was evaluated by a gastroenterologist and infectious diseases specialist to determine if the cause of death was possibly attributable to FMT.

### 2.3. Search of Literature

A literature search was performed in PubMed and Google Scholar on 14 July 2020. The following MeSH terms were used: “FMT”, “fecal microbiota transplantation”, “*Clostridium/Clostridioides* difficile infection”, “follow-up”. A total of 86 abstracts were screened. Only studies who reported on a follow-up of one year or more after FMT for rCDI specifically were included. Abstract messages without corresponding articles were excluded.

## 3. Results

### 3.1. Patients Treated with FMT

**Figure 2:**
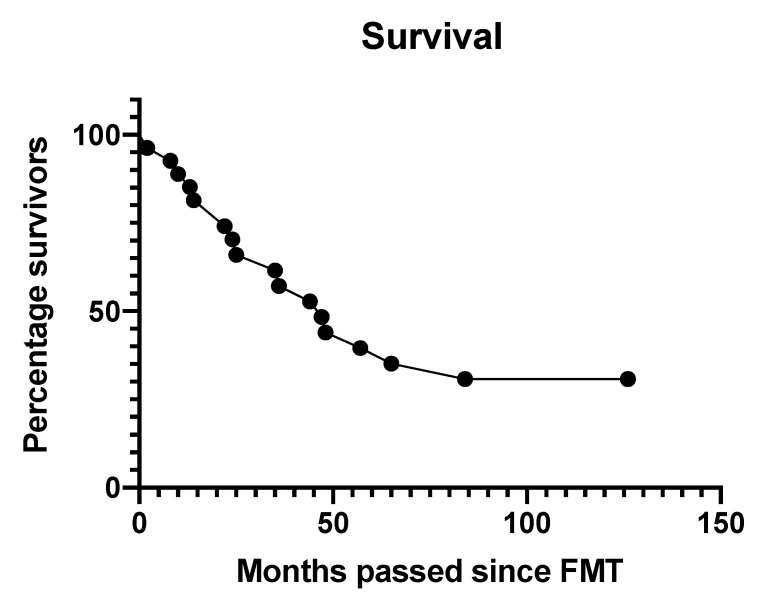
Survival following FMT of patients (*n* = 34) from the randomized controlled trial [7].

In total, 34 patients (79%) with a median age of 73 years, ranging between 18 and 101 years of age, were treated with FMT for rCDI. At two years follow-up, 25 patients were alive, 9 were deceased, and none were lost to follow-up. Ten years after completion of the trial, 7 patients were still alive, 24 had deceased, and 3 were lost to follow-up. The median follow-up of all patients was 3.5 years (range 0.2–11 years). Patient characteristics are shown in Table 1. Time of death after completion of the trial occurred after a median of 2.1 years (range 0.2–7 years) (Figure 2). Causes of death are listed in Table 2. The most commonly reported causes of death were natural causes attributed to old age and dementia, renal failure, and myocardial infarction. None of the patients died due to causes directly attributable to FMT, as evaluated by the experts. The patient who died from urosepsis had suffered from recurring urinary tract infections and urosepsis prior to FMT. However, given the time passed between the new urosepsis and FMT, it cannot be ruled out completely as being related to FMT, as no microbiological data from donor and recipient was available for evaluation of a possible causal relationship. Two patient records only stated that the patient had deceased, no cause or time of death could be retrieved. Reported malignancies, renal failure, and cirrhosis were already present at the time of inclusion in the trial. There was no indication that FMT ameliorated or deteriorated the clinical course of these pre-existing disorders. None of the patients reported the onset of a new autoimmune disorder, gastrointestinal disorder, or malignant disease after receiving FMT.

Overall, 29 (85%) patients were prescribed at least one course of non-CDI antibiotics after receiving FMT. During follow-up, four (12%) patients suffered from a new episode of CDI. All of these episodes were preceded by a course of non-CDI antibiotics. Post-FMT CDI was cured with a single course of vancomycin in all these patients. 

### 3.2. Patients Treated without FMT

Eight patients randomized to vancomycin-based treatment did not receive FMT. Of these eight patients, seven were cured with vancomycin, and one other patient refused further treatment for rCDI and died due to hospital-acquired pneumonia during the course of the study [7]. No further follow-up was performed for patients treated with vancomycin.

### 3.3. Review of Literature

A total of seven studies were found with a follow-up of at least one year or more [6,15,16,17,18,19,20], describing a total of 548 patients treated with FMT for rCDI. Results are summarized in Table 3 and Table 4. The reported follow-up ranged from one to six years. One study reports on a substantial cohort of 207 patients with a mean follow-up of 34 months [20]. Only one study had a prospective observational design with a follow-up of 12 months [16]. The other studies describe small retrospective cohorts of patients. None of the studies reported deaths directly attributable to FMT.

Adverse events can be divided into infectious complications related to the donor feces preparation used, and (theoretical) the new onset of disorders or deterioration of existing conditions in which the gut microbiota is involved in the pathogenesis. Six of the seven included studies reporting on the onset of new medical conditions post-FMT [6,16,17,18,19,20], of which five describe a prevalence of 29–51% of any new medical condition (Table 4). Only two studies reported on the deterioration of pre-existing medical conditions in 5–7% (*n* = 15) of patients [17,20]. In contrast, the amelioration of pre-existing medical conditions was 7–11% and reported/described in four studies comprising a total of 442 patients [17,18,19,20]. A specified overview of all reported conditions is shown in the “New Medical Conditions per Study” section at the end of this manuscript.

### 3.4. Outcome of rCDI

The primary cure rate of FMT was only reported in three studies [6,16,20], and was 82% to 100%. Importantly, sustained cure defined as no relapse of CDI during entire follow-up period ranged between 75% and 100%. Almost all post-FMT relapses of CDI were preceded by one or multiple courses of antibiotics. In most cases, a single course of specific antibiotics directed against CDI was sufficient to resolve CDI symptoms and prevent further recurrence of CDI. Roughly 50% of patients treated with FMT were treated with at least one reported course of antibiotics for non-CDI infections (Table 3).

### 3.5. Infectious Complications

One study provided an in-depth overview of the occurrence of infectious disorders after FMT [20]. Urinary tract infection (*n* = 31), pneumonia (*n* = 16), and sinusitis (*n* = 13) were most commonly found, in 41% (84/207) of the patients. The authors concluded that none of these infections could be directly attributed to FMT. Other studies did not report specifically about infectious complications, which probably indicates underreporting of these conditions.

### 3.6. Weight Gain

Two studies reported on changes in body weight post-FMT [17,18]. Lee et al. described weight gain (defined as >5 kg increase in body weight) in 11 patients (48%), while unchanged and decreased body weight occurred in 10 patients (44%) and 2 patients (8%), respectively [18]. The study performed by Jalanka et al. described weight gain in 76 patients (55%) [17]. Importantly, those studies do not address previous weight loss caused by recurrent CDI for which FMT is given.

### 3.7. Irritable Bowel Syndrome

The onset of irritable bowel syndrome (IBS) or IBS-like symptoms have been reported to occur in up to 25% of patients after resolution of CDI [21]. Long-term follow-up after FMT revealed the onset of IBS in 17 out of 285 (6%) patients throughout three studies reporting on IBS [17,18,20]. Of note, Jalanka et al. found a statistically significant difference (*p* = 0.034) in the onset of IBS symptoms in patients treated for CDI, favoring FMT (11%) when compared to those treated with antibiotics (36%) [17]. Furthermore, 18 patients experienced amelioration of their pre-existing IBS symptoms post-FMT [17,18,20]. However, the lack of pre-FMT patient demographics makes interpretation of these results difficult.

### 3.8. Inflammatory Bowel Disease

Four of the included studies described the effects of FMT on inflammatory bowel disease (IBD, i.e., Crohn’s disease, ulcerative colitis (UC), or undifferentiated colitis) after long-term follow-up of 423 patients [17,18,19,20]. Two patients in one study suffered from the onset of IBD during follow-up [20], none of the other studies reported new cases of IBD post-FMT [17,18,19]. A total of three patients suffered from a flare-up of their IBD (CD *n* = 2, UC *n* = 1). Furthermore, 15 patients with pre-existent IBD had either complete resolution or significant improvement of their IBD symptoms (CD, UC, and undifferentiated colitis). The lack of complete pre-FMT patient demographic data complicates the interpretation of these findings.

### 3.9. Autoimmune Disorders

A total of five cases of new-onset autoimmune disorders were reported throughout four studies describing 423 patients [17,18,19,20]. This included psoriatic arthritis, rheumatoid arthritis, osteoarthritis, thyroiditis, and hypothyroidism. Deterioration of the symptoms of pre-existing autoimmune disorders was found in three patients (unspecified *n* = 2, rheumatoid arthritis *n* = 1). In contrast, amelioration of autoimmune disorders were reported in five patients—one patient with rheumatoid arthritis (*n* = 1), one patient with alopecia areata (*n* = 1), and three unspecified cases (*n* = 3).

### 3.10. Malignancies

The onset of new malignancies post-FMT was reported in three studies [18,19,20]. A total of ten cases were identified within these studies. This included two cases of colon cancer [19,20]. Furthermore, chronic lymphocytic leukemia (*n* = 2), skin cancer (*n* = 1), breast cancer (*n* = 2), adenocarcinoma of the stomach (*n* = 2), and a neuroendocrine tumor were identified [18,20]. There were no reports of any amelioration or deterioration of pre-existing malignancies. The time frame between FMT and diagnosis of malignancies in these studies was not reported, but the relatively short follow-up interval of the studies does not seem compatible with FMT playing a causative role in carcinogenesis. In addition, no clusters of (associated) malignancies could be identified.

### 3.11. Other Disorders

Cases with new-onset non-alcoholic fatty liver disease, mastocytosis, and chronic pancreatitis were also reported. Two patients with pre-existing Parkinson’s disease had significant beneficial improvement in motor symptoms, and one patient with common variable immune deficiency experienced significantly less intermittent infections [17,19,20].

## 4. Discussion

This study is the first to report on a follow-up of up to 11 years post-FMT. Our results, in combination with a review of the available literature, suggests that FMT does not cause serious long-term adverse events. Furthermore, FMT was found to provide long-term protection against the development of a new episode of CDI, even after use of a non-CDI antibiotic. None of our relatively older patients suffered from the onset of a new autoimmune, gastrointestinal, or malignant disease following FMT, and no deaths directly attributable to FMT could be identified.

The high mortality rate among patients included in the trial is in concordance with other studies [5,22,23], and reflects the frailty of the relatively older patients suffering from rCDI, illustrated by older age (mean 72 years), comorbidity, and a decreased Karnofsky performance score at inclusion in the trial [7]. After termination of the trial, 85% of patients were exposed to a single or multiple course of antibiotics during follow-up. Interestingly, only four (12%) patients developed a new episode of CDI, and after specific antibiotic use, none of them had subsequent recurrences. Apparently, there is a true reset of the microbiota, resulting in a regained efficacy of anti-CDI antibiotics after CDI. This was also observed by van Beurden et al., who noted that FMT is not required to treat post-FMT relapses of CDI in most patients [6]. Furthermore, several other studies showed that exposure to non-CDI antibiotics post-FMT is frequent after FMT, but only rarely results in a new episode of CDI [17,18,19,20]. However, as most post-FMT recurrences of CDI are preceded by a course of antibiotics, antibiotic stewardship is warranted [6,15,16,17,18,19,20].

A limitation of our study is the relatively small sample size of 34 patients, with only 10 (21%) patients alive at the end of the follow-up period, and a lack of fecal samples to investigate whether the effects of FMT on the microbiota of patients are still present after long-term follow-up. However, the study does describe a well-defined prospective cohort, and the results do not differ from other reports [6,15,16,17,18,19,20], with a follow-up range of one to six years. So far, none of the published studies reported deaths or long-term serious adverse events directly attributable to FMT. Thus, FMT appears as a safe treatment modality without proven long-term adverse events. However, the rCDI population is relatively old, and no clear conclusions on the safety of FMT given to a younger population should be drawn. In particular, conclusions about the long-term effects of FMT in young patients on the development of malignancies, neurodegenerative, or autoimmune disorders cannot be drawn based on our study and the available literature. More prospective studies addressing the long-term follow-up after FMT are needed, preferably in national or even international registries [24]. Combining long-term follow-up data with microbiota analysis of samples of used FMT suspensions and its respective recipients in the case of adverse events may result in further understanding of the safety and long-term effects of FMT in the future.

Interestingly, several studies reported on the amelioration of other pre-existing medical conditions, such as IBS, IBD, and Parkinson’s disease after FMT [17,18,19]. In contrast, flare-ups of IBD, IBS, or IBS-like symptoms were also reported [17,18,19,20]. Whether worsening or a flare-up can be attributed to FMT or reflect the clinical course of IBD with concurrent CDI is not conclusive and therefore remains unknown. New cases of IBD after FMT are reported occasionally [20]. This may well reflect a pre-existent IBD that mimics the course of rCDI. In fact, careful consultation of FMT requests for rCDI may reveal new-onset IBD in ~10% of cases [5]. Importantly, a study reporting on the follow-up of patients treated with FMT preparations from a donor that developed IBD during follow-up did not report new-onset IBD in those patients, suggesting that transferring an IBD-prone microbiota is not sufficient to induce IBD [25]. In that context, FMT as a new treatment approach for ulcerative colitis has gained increasing interest, with FMT showing similar efficacy when compared to biologicals in pooled analysis [26]. While FMT appears partly successful as a therapy for ulcerative colitis (UC) [27], less is known about its effects on Crohn’s disease (CD). Although a positive effect of FMT on IBD is generally assumed, a meta-analysis showed that approximately 5% of IBD patients suffer from a flare-up post-FMT [28]. Ongoing studies are expected to increase our understanding of the effects of FMT on the course of IBD in the near future.

The occurrence of post-infectious IBS-like symptoms is unfortunately common after resolving CDI (~25%) [21], and IBS is also reported after FMT in 17 of 285 rCDI patients [17,18,20]. Whether IBS-like symptoms after FMT for rCDI reflect a pre-existent occurrence of IBS-like symptoms, post-infectious IBS-like symptoms after CDI, or an FMT-related phenomenon remains uncertain. The finding that post-infectious IBS after CDI is more likely to occur after antibiotic treatment compared to FMT suggests that those complaints are not caused by FMT [17]. Importantly, Perler et al. reported that 10 patients had an improvement of their pre-existing IBS after FMT for rCDI [20]. However, the available studies may be biased because the observations were mostly retrospective in nature and present a small number of patients. Therefore, further studies are needed to address the occurrence of post-infectious IBS after FMT for rCDI.

In conclusion, after a follow-up of up to 11 years of patients after inclusion in a randomized controlled trial of FMT for rCDI, long-term adverse events or complications directly attributable to FMT were not encountered. These observations are substantiated by a review of the available literature. Larger observational studies comparing appropriate controls with patients who received FMT for rCDI or other disorders are needed to confirm the long-term safety of FMT. Separate studies are needed to confirm the safety of FMT in younger patients, e.g., in patients with IBD or IBS. National or international registries collecting prospective follow-up data from all patients treated with FMT will eventually provide a more complete view on the long-term safety of FMT.

## 5. New Medical Conditions per Study

Perler et al. [20]: urinary tract infection (31), sinus infection (13), pneumonia (16), pneumonia with sepsis (2), periodontal infection (4), Mycobacterium avium complex (1), cytomegalovirus colitis (1), cryptosporidium (1), ascending cholangitis with septic shock (1), streptococcal pharyngitis (2), Lyme/ehrlichiosis/babesiosis (1), otitis media (2), Lyme disease (1), pyelonephritis (1), bronchitis (2), hordeolum (1), urinary sepsis (1), necrotizing fasciitis (1), fungal infection (2), colonic arteriovenous malformation (1), gastric ulcer (1), rectal bleed (1), unknown gastrointestinal bleed (1), diverticulitis (3), IBS (10), pancreatic insufficiency (1), small intestinal bacterial overgrowth (3), gastroesophageal reflux disease (1), IBD (2), colon resection from IBD complications (1), ulcerative colitis requiring total colectomy (1), myocardial infarction (2), chronic hypertension (1), congestive heart failure (1), atrial fibrillation (1), hyperlipidemia (1), valve replacement (1), colon cancer (1), chronic lymphocytic leukemia (2), skin cancer (1), breast cancer (1), adenocarcinoma of the stomach (2), neuroendocrine tumor (1), kidney transplant (4), nephrolithiasis (1), ovarian cyst (1), cerebrovascular accident (1), transient ischemic attack (1), chronic Alzheimer dementia (1), cognitive impairment (1), neuropathy (1), anxiety disorder (1), psoriatic arthritis (1), mastocytosis (1), and osteoarthritis (2).

Lee et al. [18]: breast cancer (mastectomy only) (1), osteoarthritis of knee (hemiarthroplasty) (1), stage 4 osteoporosis (1), transient ischemic attack (1), rheumatoid arthritis (1), hypertension (1), and IBS (2).

Mamo et al. [19]: constipation (3), diabetes mellitus (2), microscopic colitis (1), gastric ulcer (1), osteoporosis (1), non-alcoholic fatty liver disease (1), femoral avascular necrosis (1), colon cancer (1), kidney disease/end-stage renal disease (5), hypothyroidism (2), back pain (2), stroke (2), memory loss (2), arrhythmias (2), hemoptysis (1), pyoderma (1), Meniere’s disease exacerbation (1), unilateral vision loss (1), sustained ventricular tachycardia (1), peeling skin (1), arthritis (1), peanut allergy (1), narcolepsy (1), aspiration (1), dysautonomia (1), pleural effusion (1), chronic pancreatitis (1), carpal tunnel syndrome (1), benign prostatic hyperplasia (1), anxiety (1), and tonic–clonic seizure (1).

Jalanka et al. [17]: IBS (5), allergy (3), migraine (4), dementia (3), and autoimmune thyroiditis (1).

## Figures and Tables

**Table 1:** Long-term follow-up of FMT recipients from the FECAL trial.

**Inclusion**	
Number of patients:	34
Number of cured patients:	30 (88%)
Average age in years at inclusion (SD):	71.7 (11.6)
Charlson comorbidity index (SD):	2.53 (2.1)
Karnofsky performance status (SD):	51 (18)
Mean number of FMTs given:	1.26
**Follow-up**	
Number of patients still alive:	7 (21%)
Mean duration of follow-up in years (range):	4.5 (0.1–11)
Mean duration of follow-up in years (range) of patients still alive:	10.5 (9.5–11)
New episodes of CDI:	4 (12%)
Course of non-CDI antibiotics:	29 (85%)
Long-term complications of FMT:	0
Long-term complications of CDI:	1 (3%)
Lost to follow-up *:	3 (9%)
Onset of new autoimmune or gastrointestinal (GI) disorders:	0

* Three patients were healthy 2 years after FMT, but were lost to further follow-up. Abbreviations: CDI: *Clostridioides difficile* infection, FMT: fecal microbiota transplantation, SD: standard deviation.

**Table 2:** Cause of death, time between FMT and death, and possible relation with FMT from patients in the FECAL trial during long-term follow-up after FMT treatment and cure of rCDI.

Cause of Death	Number of Patients	Time Passed Since FMT (Months)	Relatable to FMT/CDI
Renal failure	3	2, 22, 25	No
Pneumonia	3	35, 48, NA	Unlikely
Stroke	2	8, 14	No
Urosepsis/sepsis	2	3, 41	Unlikely
Dementia and natural aging	3	44, 57, NA	No
Myocardial infarction	3	36, 84, NA	No
Cholecystitis	1	47	No
Cirrhosis	1	24	No
Myelodysplastic syndrome	1	22	No
Peritonitis carcinomatosa	1	65	No
Malignancy	1	10	No
CDI and peritonitis	1	13	Yes
Unclear	2	NA	NA
Total	24		

Abbreviations: CDI: *Clostridioides difficile* infection, NA: not available.

**Table 3:** Studies reporting on long-term follow-up after FMT.

Author	Study Design	Patients Treated with FMT	Deaths	Patients Included for Follow-Up	Mean Age	Mean Follow-Up	Primary Cure *	Patients with New Episodes of CDI	Patients Receiving Antibiotics Post-FMT **	Safety: SAEs Possibly Attributable to FMT
Van Beurden et al. [6].	Retrospective cohort	43	8 (19%)	39	73	21 months	82%	7 (18%)	NA	Not reported
Perler et al. [20].	Retrospective cohort	528	52 (10%)	207	58	34 months	89%	51 (25%)	100 (48%)	Not reported
Lee et al. [18].	Retrospective cohort	94	37 (39%)	23	NA	6 years	NA	0	12 (52%)	Not reported
Girotra et al. [16].	Prospective observational cohort	29	0	29	80	12 months	100%	0	NA	Not reported
Mamo et al. [19].	Retrospective cohort	232	26 (11%)	137	66	22 months	NA	24 (18%)	61 (45%)	Not reported
Greenwald et al. [15].	Retrospective cohort	79	0	58	69	18 months	NA	11 (19%)	NA	Not reported
Jalanka et al. [17].	Retrospective cohort	NA	0	55	57	3.4 years	NA	2 (4%)	26 (47%)	Not reported

* Primary cure is defined as a resolution of CDI symptoms for 48 consecutive hours post-FMT. ** At least one course of non-CDI antibiotics. Abbreviations: CDI: *Clostridioides difficile* infection, FMT: fecal microbiota transplantation, NA: not available, SAEs: serious adverse events.

**Table 4:** Studies reporting on other medical conditions post-FMT.

Author	Onset of New Medical Condition * Patients (%)	Deterioration of Medical Condition ** Patients (%)	Amelioration of Medical Condition ** Patients (%)
Perler et al. [20]	105 (51%)	11 (5%)	15 (7%)
	Infectious disorders: *n* = 84		
	AD: *n* = 3	Rheumatoid arthritis: *n* = 1	IBS: *n* = 10
	Malignancies: *n* = 8		IBD: *n* = 4
	IBD/IBS: *n* = 12	IBD: *n* = 10	Alopecia areata: *n* = 1
	Other: *n* = 33		
Lee et al. [18]	8 (35%)	Not available	7 (30%)
	Infectious disorders: *n* = 0		IBD: *n* = 4
	AD: *n* = 1		Diabetes mellitus: *n* = 1
	Malignancies: *n* = 1		Parkinson’s disease: *n* = 2
	IBD/IBS: *n* = 2		
	Other: *n* = 4		
Mamo et al. [19]	43 (31%)	Not available	12 (7%)
			Rheumatoid arthritis: *n* = 1
	Infectious disorders: *n* = 1		IBS: *n* = 3
	Autoimmune disorders: *n* = 0		IBD: *n* = 2
	Malignancies: *n* = 1		Diverticulosis: *n* = 2
	IBD/IBS: *n* = 0		Diabetes mellitus: *n* = 1
	Other: *n* = 40		CVID: *n* = 1
Jalanka et al. [17]	16 (29%)	4 (7%)	8 (11%)
	Infectious disorders: *n* = 0		
	AD: *n* = 1	Diabetes mellitus: *n* = 2	IBD: *n* = 5
	Malignancies: *n* = 0		
	IBD/IBS: *n* = 5	AD: *n* = 2	AD: *n* = 3
	Other: *n* = 10		

* All new-onset medical conditions reported in footnote. ** Medical conditions present before FMT. Abbreviations: AD = autoimmune disease, CVID: common variable immunodeficiency, IBD = inflammatory bowel disease, IBS = irritable bowel disease.

## Data Availability

Data is available with corresponding authors upon reasonable request.

## References

[1] Deshpande A., Pasupuleti V., Thota P., Pant C., Rolston D.D.K., Hernandez A.V., Donskey C.J., Fraser T.G. (2015). Risk Factors for Recurrent Clostridium difficile Infection: A Systematic Review and Meta-Analysis. Infect. Control Hosp. Epidemiol..

[2] Debast S.B., Bauer M.P., Kuijper E.J. (2014). European Society of Clinical Microbiology and Infectious Diseases: Update of the treatment guidance document for Clostridium difficile infection. Clin. Microbiol. Infect..

[3] McDonald L.C., Gerding D.N., Johnson S., Bakken J.S., Carroll K.C., Coffin S.E., Dubberke E.R., Garey K.W., Gould C.V., Kelly C. (2018). Clinical Practice Guidelines for Clostridium difficile Infection in Adults and Children: 2017 Update by the Infectious Diseases Society of America (IDSA) and Society for Healthcare Epidemiology of America (SHEA). Clin. Infect. Dis..

[4] Ooijevaar R.E., van Beurden Y.H., Terveer E.M., Goorhuis A., Bauer M.P., Keller J.J., Mulder C.J.J., Kuijper E.J. (2018). Update of treatment algorithms for Clostridium difficile infection. Clin Microbiol. Infect..

[5] Terveer E.M., Vendrik K.E., Ooijevaar R.E., Lingen E.V., Boeije-Koppenol E., Nood E.V., Goorhuis A., Bauer M.P., van Beurden Y.H., Dijkgraaf M.G. (2020). Faecal microbiota transplantation for Clostridioides difficile infection: Four years’ experience of the Netherlands Donor Feces Bank. United Eur. Gastroenterol. J..

[6] Van Beurden Y.H., de Groot P.F., van Nood E., Nieuwdorp M., Keller J.J., Goorhuis A. (2017). Complications, effectiveness, and long term follow-up of fecal microbiota transfer by nasoduodenal tube for treatment of recurrent Clostridium difficile infection. United Eur. Gastroenterol. J..

[7] Van Nood E., Vrieze A., Nieuwdorp M., Fuentes S., Zoetendal E.G., de Vos W.M., Visser C.E., Kuijper E.J., Bartelsman J.F., Tijssen J.G. (2013). Duodenal infusion of donor feces for recurrent Clostridium difficile. N. Engl. J. Med..

[8] Ianiro G., Maida M., Burisch J., Simonelli C., Hold G., Ventimiglia M., Gasbarrini A., Cammarota G. (2018). Efficacy of different faecal microbiota transplantation protocols for Clostridium difficile infection: A systematic review and meta-analysis. United Eur. Gastroenterol. J..

[9] Ooijevaar R.E., Terveer E.M., Verspaget H.W., Kuijper E.J., Keller J.J. (2019). Clinical Application and Potential of Fecal Microbiota Transplantation. Annu. Rev. Med..

[10] Baxter M., Colville A. (2016). Adverse events in faecal microbiota transplant: A review of the literature. J. Hosp. Infect..

[11] Wang S., Xu M., Wang W., Cao X., Piao M., Khan S., Yan F., Cao H., Wang B. (2016). Systematic review: Adverse events of fecal microbiota transplantation. PLoS ONE.

[12] Terveer E.M., van Beurden Y.H., Goorhuis A., Seegers J., Bauer M.P., van Nood E., Dijkgraaf M.G.W., Mulder C.J.J., Vandenbroucke-Grauls C., Verspaget H.W. (2017). How to: Establish and run a stool bank. Clin. Microbiol. Infect..

[13] DeFilipp Z., Bloom P.P., Torres Soto M., Mansour M.K., Sater M.R.A., Huntley M.H., Turbett S., Chung R.T., Chen Y.B., Hohmann E.L. (2019). Drug-Resistant E. coli Bacteremia Transmitted by Fecal Microbiota Transplant. N. Engl. J. Med..

[14] Bunnik E.M., Aarts N., Chen L.A. (2017). Physicians Must Discuss Potential Long-Term Risks of Fecal Microbiota Transplantation to Ensure Informed Consent. Am. J. Bioeth..

[15] Greenwald T.P.D., Mcquillen D., Barto A. (2016). Colonoscopy-assisted Fecal Microbiota Transplant for Outpatient Treatment of Recurrent or Refractory Clostridium Difficile Colitis; Long Term Follow-up of 58 Patients. J. Clin. Gastroenterol. Treat..

[16] Girotra M., Garg S., Anand R., Song Y., Dutta S.K. (2016). Fecal Microbiota Transplantation for Recurrent Clostridium difficile Infection in the Elderly: Long-Term Outcomes and Microbiota Changes. Dig. Dis. Sci..

[17] Jalanka J., Hillamaa A., Satokari R., Mattila E., Anttila V.J., Arkkila P. (2018). The long-term effects of faecal microbiota transplantation for gastrointestinal symptoms and general health in patients with recurrent Clostridium difficile infection. Aliment Pharmacol. Ther..

[18] Lee C.H., Chai J., Hammond K., Jeon S.R., Patel Y., Goldeh C., Kim P. (2019). Long-term durability and safety of fecal microbiota transplantation for recurrent or refractory Clostridioides difficile infection with or without antibiotic exposure. Eur. J. Clin. Microbiol. Infect. Dis..

[19] Mamo Y., Woodworth M.H., Wang T., Dhere T., Kraft C.S. (2018). Durability and Long-term Clinical Outcomes of Fecal Microbiota Transplant Treatment in Patients With Recurrent Clostridium difficile Infection. Clin. Infect. Dis..

[20] Perler B.K., Chen B., Phelps E., Allegretti J.R., Fischer M., Ganapini V., Krajiceck E., Kumar V., Marcus J., Nativ L. (2020). Long-Term Efficacy and Safety of Fecal Microbiota Transplantation for Treatment of Recurrent Clostridioides difficile Infection. J. Clin. Gastroenterol..

[21] Wadhwa A., Al Nahhas M.F., Dierkhising R.A., Patel R., Kashyap P., Pardi D.S., Khanna S., Grover M. (2016). High risk of post-infectious irritable bowel syndrome in patients with Clostridium difficile infection. Aliment Pharmacol. Ther..

[22] Olsen M.A., Stwalley D., Demont C., Dubberke E.R. (2019). Clostridium difficile infection increases acute and chronic morbidity and mortality. Infect. Control Hosp. Epidemiol..

[23] Shorr A.F., Zilberberg M.D., Wang L., Baser O., Yu H. (2016). Mortality and Costs in Clostridium difficile Infection Among the Elderly in the United States. Infect. Control Hosp. Epidemiol..

[24] Keller J.J., Ooijevaar R.E., Hvas C.L., Terveer E.M., Lieberknecht S.C., Hogenauer C., Arkkila P., Sokol H., Gridnyev O., Megraud F. (2020). A standardised model for stool banking for faecal microbiota transplantation: A consensus report from a multidisciplinary UEG working group. United Eur. Gastroenterol. J..

[25] Fischer M., Bittar M., Papa E., Kassam Z., Smith M. (2017). Can you cause inflammatory bowel disease with fecal transplantation? A 31-patient case-series of fecal transplantation using stool from a donor who later developed Crohn’s disease. Gut Microbes.

[26] Zhou H.Y., Guo B., Lufumpa E., Li X.M., Chen L.H., Meng X., Li B.Z. (2020). Comparative of the Effectiveness and Safety of Biological Agents, Tofacitinib, and Fecal Microbiota Transplantation in Ulcerative Colitis: Systematic Review and Network Meta-Analysis. Immunol. Investig..

[27] Costello S.P., Soo W., Bryant R.V., Jairath V., Hart A.L., Andrews J.M. (2017). Systematic review with meta-analysis: Faecal microbiota transplantation for the induction of remission for active ulcerative colitis. Aliment Pharmacol. Ther..

[28] Qazi T., Amaratunga T., Barnes E.L., Fischer M., Kassam Z., Allegretti J.R. (2017). The risk of inflammatory bowel disease flares after fecal microbiota transplantation: Systematic review and meta-analysis. Gut Microbes.

